# Preparation and Properties of Highly Electroconductive and Heat-Resistant CMC/Buckypaper/Epoxy Nanocomposites

**DOI:** 10.3390/nano8120969

**Published:** 2018-11-24

**Authors:** Ting Zheng, Guanhui Wang, Nuo Xu, Chunrui Lu, Yingjie Qiao, Dongxing Zhang, Xiaodong Wang

**Affiliations:** 1 School of Materials Science and Technology, Harbin Institute of Engineering, Harbin 150001, China; zthappy1127@gmail.com (T.Z.); xunuo_hit@163.com (N.X.); luchunrui06@126.com (C.L.); 2 School of Chemistry and Chemical Engineering, Jinggangshan University, Ji’an 343009, China; guanhuiwang2011@gmail.com; 3 College of Materials Science and Chemical Engineering, Harbin Engineering University, Harbin 150001, China; qiaoyingjie99@163.com

**Keywords:** cellulose, buckypapers, nanocomposites, electrical properties, mechanical properties, high-temperature properties

## Abstract

Self-assembled buckypapers have been successfully prepared using sodium carboxyl methyl cellulose (CMC) as a binder. The lowest resistivity that was reached was 0.43 ± 0.03 Ω·m, when the buckypapers were prepared by the same mass of CMC and carboxy-modified carbon nanotubes (CNTs). A heat-resistant electroconductive nanocomposite with epoxy resin as the matrix and CMC/buckypapers as the reinforcement was fabricated by a resin impregnation molding technique. The effects of CMC/buckypaper layers on the conductivity, thermal stability, and mechanical and dynamic mechanical performance of the epoxy resin polymer nanocomposites were investigated. It was found that CMC/buckypapers hold great promise for improving the properties of nanocomposites, and the buckypapers’ performance can be enhanced by using modified CNTs to prepare them. The obtained nanocomposites showed an approximate 71.23% bending strength improvement (125.04 ± 5.62 MPa) and a 30.71% bending modulus improvement (5.83 ± 0.68 GPa), with an increased number of CMC/buckypaper layers. An enhanced degradation temperature and residual mass were also achieved for the nanocomposites when compared with a pure polymer. The nanocomposites with four CMC/buckypaper layers possessed the highest storage modulus (1934 MPa), which was approximately 60% higher than that of a neat polymer (1185 MPa). Therefore, CMC/buckypapers could be effectively used to manufacture heat-resistant electroconductive polymer nanocomposites with improved properties.

## 1. Introduction

Electrically conductive polymer composites have received considerable attention, owing to their multifunctional applications in many engineering and electronic fields, such as aerospace, electromagnetic shielding, and electronics, among others [1,2,3]. Epoxy resins are the most important thermosetting polymers [4,5]. With good compatibility, excellent mechanical properties, thermal stability, chemical resistance, and flame retardancy, epoxy resins have been widely used in the aerospace industry, surface coatings, electrical and optical practices, and matrices in high-heat-resistant performance composites [6], and have become a good candidate material for electrically conductive polymer composites. The modification of epoxy resins is an effective method of endowing material with an electrically conductive function and alternating the drawbacks of epoxy resins, such as their brittleness and poor resistance to crack propagation [7]. Conventionally, metal powders, such as Al, Au, Ag, and Cu, have been widely used to fabricate electrically conductive polymer composites. However, these kinds of metal-based composites have several weak points, such as easy oxidization, emission of electromagnetic waves, and high manufacturing costs. Therefore, polymer composites containing carbonaceous fillers (CNTs, graphene and carbon nanofibers) have attracted a large amount of interest for preparing electrically conductive polymer materials. In addition, these kinds of electrically conductive materials offer advantages of being lightweight, corrosion resistant, easy to process, and low cost [8].

Carbon nanotubes are widely used as reinforcement materials for polymer composites due to their high specific surface area and their remarkable mechanical and electrical conductivity properties [9,10,11]. However, the dispersion of CNTs into a polymer matrix is extremely difficult due to their strong agglomerating properties, low solubility in polymer matrices, and high viscosity of resins during fabrication [12,13]. This limits not only the ability to exploit the advantages of CNTs, but also their practical applications. Therefore, individual CNTs have often been assembled into micro/macro structures, such as membranes, fibers, and yarns, to promote their application in recent years [14]. Among these structures, CNT membranes, also known as “buckypapers”, are a macrostructure material which possesses unusual multifunctionalities in combination with its satisfactory electrical, significantly higher specific modulus and specific strengths [15]. More importantly, buckypapers can be easily infused with resin and manufactured into conventional composites to realize conductive/structural multifunctional composites [16]. Unlike composites with CNTs dispersed in a polymer matrix, buckypaper-based composites can avoid nanotube agglomeration to achieve a better dispersion, a much higher concentration of CNTs, and high conductive tube networks to maximize their electrical conductivity and mechanical properties [17].

Several methods of fabricating buckypapers have been developed. Among them, the vacuum filtration method is the most popular one used in research labs and industries, due to its simplified operational procedure, availability of raw materials, and moderate cost [18]. In this method, a suspended solution containing CNTs is dispersed using surfactants and then filtered by a vacuum filtration bump to induce paper formation [19,20]. This remarkable buckypaper structure, however, should still be very carefully handled during preparation. Furthermore, until recently, it could not show the full mechanical and electrical advantages of CNTs. This was ascribed to weak van-der-Waals, poor network joints, and the lack of effective stress and current transfer among entangled carbon nanotubes [21]. To address these problems, introducing a binder into buckypapers is a reliable way to improve its mechanical and electrical properties.CMC is a representative cellulose derivative with tasteless, nontoxic, and water-soluble characteristics, which makes it widely applied in flocculation, stabilizing, oil drilling, food processing, drag reduction, and detergents [22]. CMC is especially used in paper making and works as a bonding agent in textiles [23], so it is an ideal material to improve the performance of buckypapers and to help carbon nanotubes to form membranes.

In this study, a self-assembled electroconductive buckypaper with low resistance was prepared by adding CMC as a binder. Four types of buckypapers without and with CMC were prepared using modified and unmodified CNTs as materials. The morphologies, resistivity, and Raman spectra were used for testing and analyzing. In addition, we developed a highly conductive and thermally stable nanocomposite for heat-resistant applications, using epoxy resin as the matrix and self-assembled CMC/buckypaper as the reinforcement. The effects of CMC/buckypaper layers on the conductivity, mechanical properties, thermal stability, and dynamic mechanical performance of epoxy resin polymer nanocomposites were investigated. The results indicated that CMC/buckypaper/epoxy nanocomposites with good mechanical properties and an excellent thermal and thermomachanial performance are a promising electroconductivity material for high-temperature-resistant material applications.

## 2. Materials and Methods

### 2.1. Materials

Multi-walled CNTs (MWCNTs)and carboxyl group functionalized MWCNTs made by CVD method were received in powder with outer diameters <8 nm and lengths of 10–30 μm from TimesNano Company (Chengdu, China). Their purity was >95%. Distilled water was used as a solvent. Polyoxyethylene octylphenylether (TritonX-100, Biochemical grade, Aladdin Chemical Reagents Co., Ltd., Shanghai, China) was employed as a nonionic surfactant in fabricating CNTs. CMC was chosen as the film forming materials.

Epoxy resin of TDE85 (4,5-epoxyclyclohexyl-1,2-diglycidyldiformate, epoxy value, 0.85) was provided by Tianjin Synthetic Materials Research Institute (Tianjin, China). The m-phenylenediamine (MPD) supplied by Beijing Chemical Reagent Co. (Beijing, China) were used as curing agents.

### 2.2. Manufacturing of Buckypapers and Buckypaper Nanocomposites

**Figure 1:**
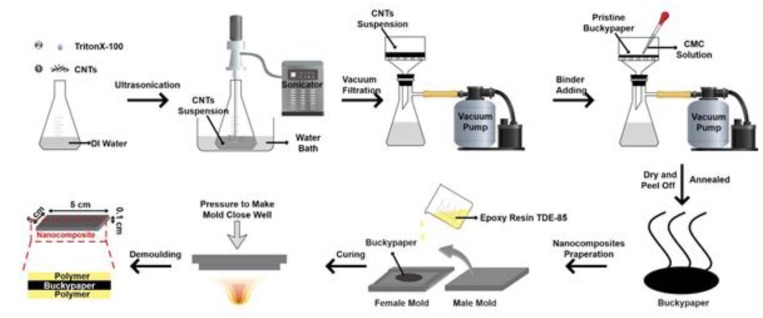
A schematic illustration of the fabrication of CMC/buckypaper/epoxy nanocomposites.

CMC was immersed into 50 mL distilled water and stirred at 80 °C for 1 h to get a good binder solution. MWCNTs were also immersed in 50 mL distilled water with the addition of 0.5 wt % Triton X-100 and sonicated at room temperature for 30 min using an ultrasonic homogenizer from Palmer Instruments (Model: CP 130 PB) to obtain a well-dispersed MWCNT suspension. Then, the suspension was filtered through a vacuum filtration setting to get a buckypaper. After the solution filtration, additional water was used to wash away the residual surfactant of buckypaper. CMC solution was added into the vacuum filtration and filtered to go through the carbon nanotube network to form self-assembled CMC/buckypapers. The obtained self-assembled buckypaper with the filter paper was vacuum-dried at 80 °C overnight and then separated. Due to its hydrophobic nature, the free-standing buckypaper was easy to peel off from the hydrophilic filter paper. The obtained free-standing buckypaper was then annealed under 120 °C for 1 h in order to remove any residual moisture. The fabrication progress is shown in Figure 1.

A resin impregnation molding technique was applied to make buckypaper-reinforced epoxy resin nanocomposites. During the fabrication process, one-layered, two-layered, three-layered, and four-layered buckypapers were first, in that order, placed on the bottom of the female mold. An amount of thermosetting epoxy resin was poured to fill the mold, and vacuumed for 30 min at 40 °C to remove the air bubbles in the epoxy resin. Then, the male mold was put on the female mold, and we used screws to close the mold completely and applied pressure to the mold to achieve the same thickness for each of the samples. Finally, thermosetting epoxy resins were cured at 120 °C in a vacuum for 6 h to produce the final nanocomposites.

### 2.3. Characterization of Buckypapers and CMC/Buckypaper Nanocomposites

#### 2.3.1. The Structure of Buckypapers and Buckypaper Nanocomposites

The morphology and microstructure of self-assembled buckypapers were observed using Scanning electron microscopy (SEM, S-4700, Hitachi Ltd.,Tokyo, Japan) with an operating voltage of 10 kV. The atomic force microscope (AFM, Nanoscope IIIa) is a kind of instrument used to study the surface structure of both conductor and nonconductive materials. Here, we used the atomic force microscope of Bruker AXS to observe the buckypaper topography over recorded images. The roughness of the buckypapers was studied by scanning the AFM probe over the sheet surface with similar conditions to those mentioned above. AFM was operated in the tapping mode, scan rate was set at 125 Hz, and scan size was 10 × 10 × 5 µm. Raman spectroscopy analysis was performed to investigate the individual structures of buckypaper, using a Raman spectrometer (XploRA, HORIBA Jobin Yvon, Paris, France) equipped with an integral microscope (Olympus BH2-UMA, Olympus, Tokyo, Japan).

#### 2.3.2. Electrical Characterization of Buckypapers

Electrical conductivities were measured using the four-point probe method. The buckypapers were cut into 5 × 3 cm rectangular strips. Four electrode pins were pressed to the paper surface with a spacing of 1 cm. Silver paste was placed at the contacting points between the paper and the electrode pins in order to eliminate contact resistance.

#### 2.3.3. Mechanical Testing

The flexural properties were characterized by three-point bending tests according to ASTM D7264 standard. Experiments are carried out using a mechanical tester machine (WDW-100L, Yangzhou, China) with a cell load capacity of 100 kN and load resolution of 0.0001 kN. The dimension of the specimens used for test was 40 × 13 × 1 mm (length, width, and thickness, respectively), the optional span-to-thick ratio of 32:1 was used and the constant deformation rate was maintained at 2 mm/min. The tests are continued until specimen failure. The bending stress and bending modulus were obtained by Equation (1) and (2):

(1) $$\sigma_{t}=\frac{3PL}{2bh^{2}}$$

(2) $$E_{t}=\frac{L^{3}m}{4bh^{3}}$$

where $\sigma_{t}$ is the bending stress (MPa), *P* is the maximum force (N), *L* is span length (mm), *b* is the width of samples (mm), and *h* is the thickness of samples (mm), $E_{t}$ is the bending modulus(MPa), m is slope of the secant of the force-deflection curve.

#### 2.3.4. Thermal Analysis

The thermal stability of nanocomposites was analyzed using thermogravimetric analysis (TGA, TG209, NETZSCH, Selb, Germany). The samples were heated from 20 °C to 700 °C with a heating rate of 20 °C/min in a nitrogen flow of 50 mL/min. Approximately 10 mg of the samples was used in the TGA test.

#### 2.3.5. Dynamic Mechanical Analysis

The thermomechanical performance of buckypapers/epoxy nanocomposites was investigated using dynamic mechanical analysis (DMA, DMA242, NETZSCH Instruments Co., Selb, Germany). The measurements were performed at a fixed frequency of 1 Hz in a three-point bending mode. The dimension of the specimens used for the test was 40 × 10 × 1 mm and the specimens were heated from 20 °C to 250 °C, with a heating rate of 5 °C/min.

## 3. Results and Discussion

### 3.1. The Morphology and Structure of Self-Assembled Buckypaper

**Figure 2:**
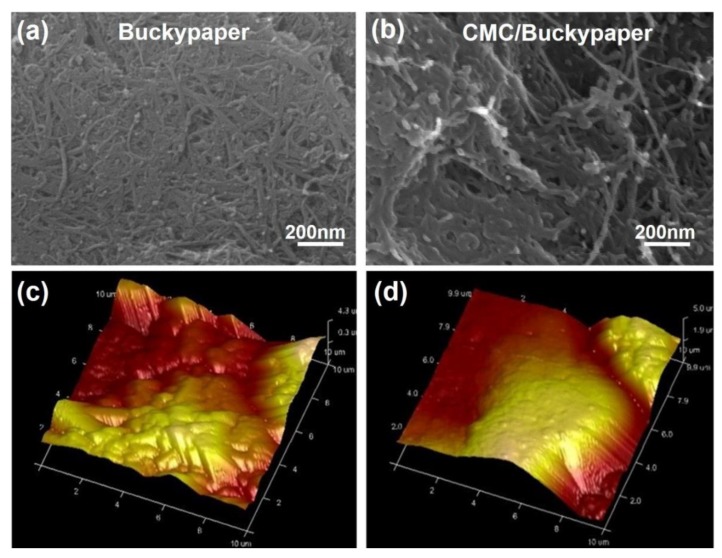
The morphology and structure of a buckypaper. (**a**) SEM picture of buckypaper; (**b**) SEM picture of CMC/buckypaper; (**c**) atomic force microscope (AFM) picture of buckypaper; (**d**) AFM picture of CMC/buckypaper.

The morphology of self-assembled buckypapers and buckypapers with a CMC binder was examined through SEM and AFM; typical images are displayed in Figure 2. The buckypaper sheet (Figure 2a) shows a great number of long and random CNTs entangle with each other very well; the CNTs appear to be homogeneously dispersed and to have formed a self-supported network structure due to interbundle Van der Waals forces and mechanical interlocking within the sheet. There were many different sizes of pores in the network and the average size was about 80 nm. Such a continuous network made of individual carbon nanotubes will act as a conductive path for electrons and make buckypapers electrically conductive. Figure 2b shows that the morphology of buckypaper treated with CMC has no obvious distinction from that of a buckypaper without a binder to compare with Figure 2a. However, it is observed from Figure 2c,d that buckypaper will be flatter and not easy to crack after CMC is introduced as a binder. CMC has a filming function, so it could help carbon nanotubes to connect with each other better and make the buckypaper more uniform. Carbon nanotube bundles were surrounded by the binder, which had uniformly penetrated through the entire buckypaper in all directions.

### 3.2. The Conductivity of Self-Assembled Buckypapers and CMC/Buckypapers

**Figure 3:**
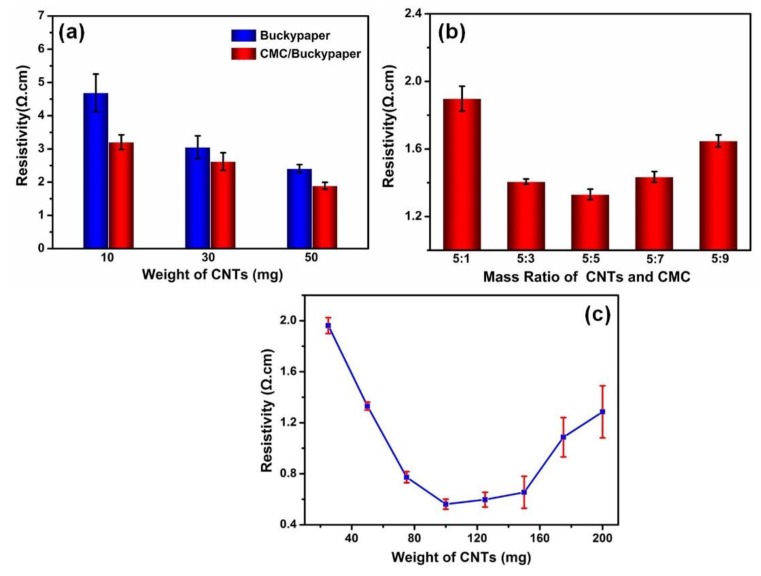
The resistivity of buckypaper (**a**) adding CMC as a binder; (**b**) the different mass ratio of carbon nanotubes (CNTs) and CMC; (**c**) the different weight of CNTs.

Regarding electrical properties, the resistivity of pristine self-assembled buckypapers and buckypapers with CMC as a binder is shown in Figure 3. The electrical conductivity of buckypapers is much smaller than that of individual CNTs, for electrical conductivity is largely limited by the intertube contact resistance in CNT networks. CMC is always used in surface sizing to increase the surface strength of paper in the paper industry, and buckypaper is actually a porous structure. Thus, CMC will penetrate buckypapers to improve the contact of carbon nanotubes when it is used as the binder in the process of making buckypapers. As shown in Figure 3a, the resistivity of the buckypaper decreased significantly after CMC was added as a binder. Just 1 mg CMC was added into the distilled water to create a binder solution, and it improved the conductivities of three different buckypapers to varying degrees.

To use CMC in a better way, the best mass ratio of CNTs and CMC was studied, as shown in Figure 3b. Fifty milligrams of CNT were immersed in 50 mL distilled water and filtered to get a buckypaper; a different weight of CMC was immersed in distilled water and filtered through buckypapers. The resistivity of the CMC/buckypaper rose after an initial decline; the lowest resistivity was 1.3 ± 0.03 Ω·cm when the mass ratio of CNT and CMC was 1:1. This is because more conductive paths were formed in the buckypaper network when we added an appropriate binder. The CMC helped carbon nanotubes to form a large and stable network; these networks provided paths for the electron transfer, which led to the reduction of the intertube contact resistance. However, the CMC binder increased the carbon nanotubes’ contact points when the surplus binder wrapped most of the carbon nanotubes. Since the binder could not transfer the electrons effectively, electrical conductivity was decreased.

Self-assembled buckypapers made with a different weight of CNTs were also investigated; the results are shown in Figure 3c. The resistivity of CMC/buckypaper was drastically improved when the CNTs’ weight was increased from 25 mg to 100 mg; however, it then exhibited an increasing trend after 125 mg. This change can be explained by a number of reasons. Firstly, some of CNTs appeared to be wrapped with a thin CMC coating, as Figure 3b shows. At the same time, the more CNTs buckypapers had, the more CMC they needed. It is generally known that materials made up of nonconducting materials and conducting carbon nanotubes exhibit a significant increase in conductivity to form electrical pathways through the material, i.e., the so-called percolation threshold [24]. For buckypapers, a lot of overlapping carbon nanotubes form good connecting electrical pathways with the increasing weight of CNTs. However, the resistance of buckypapers is controlled by the resistance of the junctions between overlapping CNTs. Therefore, it is most probably the case that too much CMC may cause an increasing junction resistance in the CNT network.

**Figure 4:**
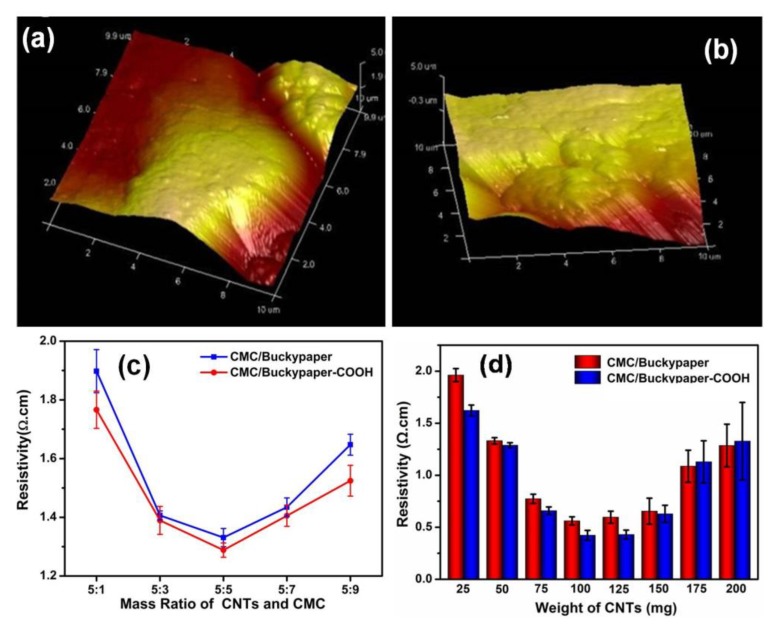
The CMC/buckypapers made with CNTs–COOH. (**a**) AFM picture of CMC/Buckypaper; (**b**) AFM picture of CMC/Buckypaper–COOH; (**c**) the resistivity of CMC/Buckypaper and CMC/Buckypaper–COOH with a different mass ratio of CNTs and CMC; (**d**) the resistivity of CMC/Buckypaper and CMC/Buckypaper–COOH with a different weight of CNTs.

The application of CNTs is restricted due to their poor solubility in most solvents. The challenge of solubilizing CNTs can be addressed through their covalent modification, such as carboxy modification. Thus, the self-assembled CMC/buckypapers made with CNTs–COOH were also studied, as Figure 4 shows. According to the results, the CMC/buckypaper–COOH was shown to be smoother and neater than the previous CMC/buckypaper, and the resistivity of the CMC/buckypaper–COOH was also improved. The lowest resistivity reached 1.26 ± 0.04 Ω·cm, when the buckypaper was prepared by the same mass of CMC and carboxy modification of carbon nanotubes, as shown in Figure 4c. A high degree of modification will disturb the p-electron conjugation of the nanotubes, and result in a reduced conductivity of the samples, but the resistivity of our samples was improved. This was probably caused by a better distribution of nanotubes in the solution, which overcompensated the influence of structural defects caused by the modification. This means that the dispersion of the CNT solution is a very important factor in the conductivity of buckypapers. CMC/buckypaper–COOH made with a different weight of CNTs were also investigated; the results are shown in Figure 4d. They showed the same trend as CMC/buckypapers and the lowest resistivity they could reach was 0.43 ± 0.03 Ω·cm.

### 3.3. The Raman Spectra Analysis of Buckypaper and CMC/Buckypaper

**Figure 5:**
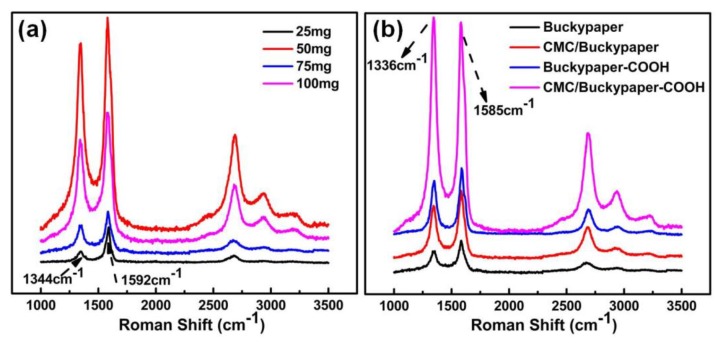
Raman spectra recorded for (**a**) a buckypaper with different CNTs weight and (**b**) different kinds of buckypaper.

The Raman spectra of the obtained self-assembled buckypapers at room temperature are shown in Figure 5. Figure 5a shows the high frequency Raman spectra for buckypapers with different CNT weight. All spectra consist of two major Raman bands, the weak D and intense G band. In a typical Raman spectrum of CNTs, the D band is labeled as the structural defect mode, which is attributed to disordered carbon and defects in the graphite crystal lattice, while the G peak reflects the crystalline ordering of the graphitic basal-plane. The D-band and G-band frequency of buckypapers was around 1344 cm^−1^ and 1592 cm^−1^, respectively, which were in agreement with literature results for CNTs [25]. A lower D/G ratio of band intensity indicates fewer defects and a higher degree of graphitic crystallinity. The D/G ratios presented a decreasing trend with an increasing amount of CNTs from 25 mg, as shown in Figure 5a. This suggests a high quality of nanotubes and buckypapers.

The spectrum of the CMC/buckypaper is essentially similar to that of the bare buckypaper, although with slightly wider and intense signals. The same trend could also be observed in the buckypaper–COOH and CMC/buckypaper–COOH. Notably, the D/G ratios of buckypapers will increase after adding CMC as a binder, as shown in Table 1. The CMC coating causes a high degree of disorder/large number of defects on the buckypaper surface due to exposed CMC at the surface, as well as additional defects within the buckypaper sheets. This is a benefit of epoxy resin impregnation when we use buckypapers to prepare electrically conductive polymer composites.

### 3.4. Electrical Resistivity of CMC/Buckypaper/Epoxy Nanocomposites

**Figure 6:**
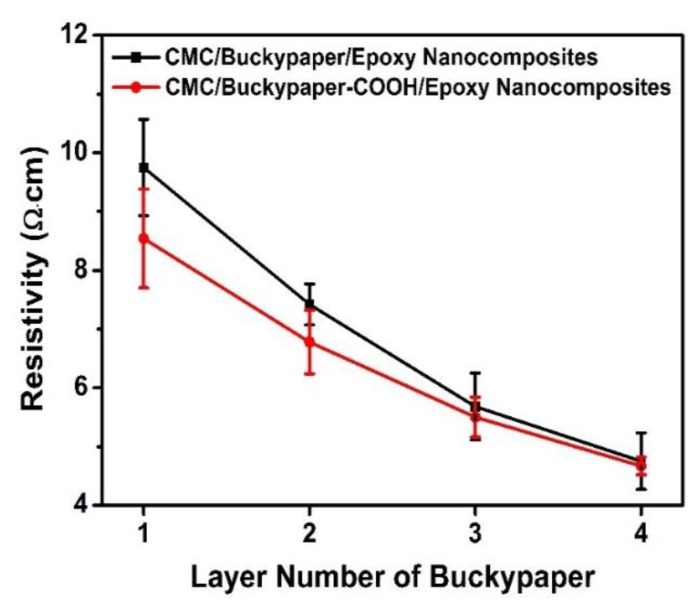
The resistivity of buckypaper nanocomposites.

The resistivity of a self-assembled CMC/buckypaper and corresponding carboxylated nanocomposites are plotted in Figure 6 as a function of buckypaper layers for those samples at room temperature. With the increase of buckypaper layers, the resistivity decreased monotonically, following a steady state tendency for both CMC/buckypaper nanocomposites and CMC/buckypaper–COOH nanocomposites. Nanocomposites reinforced with CMC/buckypaper–COOH showed lower resistivity duo to the better conductivity of buckypaper made with carboxylated carbon nanotubes; the lowest resistivity was 4.75 ± 0.15 Ω.cm. The nanocomposites that exhibit excellent electrical conductivity could be promising materials for high-performance intelligent materials, such as electromagnetic shielding and electrically driven and lightning protection applications.

### 3.5. Mechanical Property of CMC/Buckypaper/Epoxy Nanocomposites

**Figure 7:**
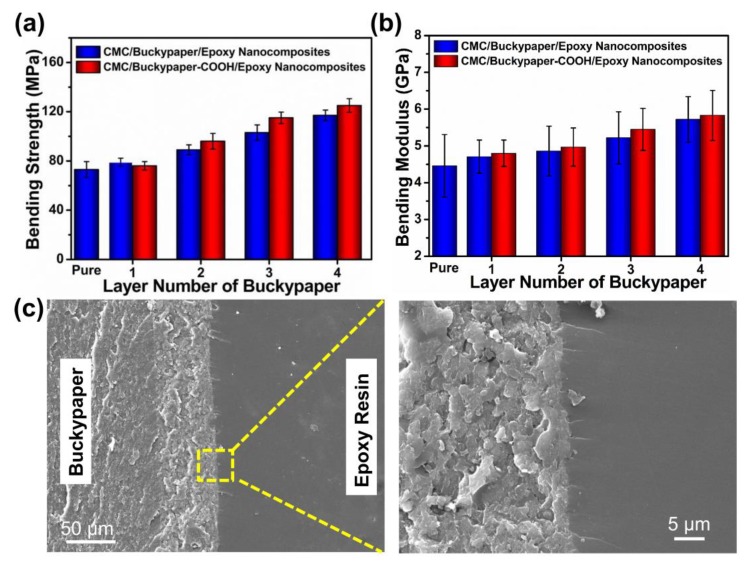
The mechanical properties of buckypaper nanocomposites. (**a**) bending strength; (**b**) bending modulus; (**c**) SEM pictures of fracture surfaces after bending test.

The mechanical properties of nanocomposites are a crucial parameter for their practical applications. Figure 7a,b shows a comparison of the bending properties for nanocomposites with different buckypaper layers. As expected, the mechanical properties of the nanocomposites exhibited had an ascending tendency with an increase in buckypaper layers, due to interfacial interaction, efficiently transfer of load and energy, reduction in stress concentration, and dissipating energy. The bending modulus varies from 4.46 ± 0.85 GPa for pure polymer to 5.83 ± 0.68 GPa for four-layered buckypaper nanocomposites, while the tensile strength increases from 73.14 ± 6.51 MPa to 125.04 ± 5.62 MPa. The typical cross-sectional fracture surfaces of nanocomposites incorporated with self-assembled buckypaper after bending testing are displayed in Figure 7c. The epoxy resin matrix showed features of brittle fracture. The area of buckypaper is distinct from epoxy resin matrix, indicating that there is interfacial bonding between the two phases. The enlarged SEM image of the interface between buckypaper and epoxy resin matrix reveals that polymer resin is evenly impregnated throughout the buckypaper at the interface. It also indicates a strong interaction and compatibility between buckypaper and epoxy resin matrix.

### 3.6. The Thermal Stability of CMC/Buckypaper/Epoxy Nanocomposites

**Figure 8:**
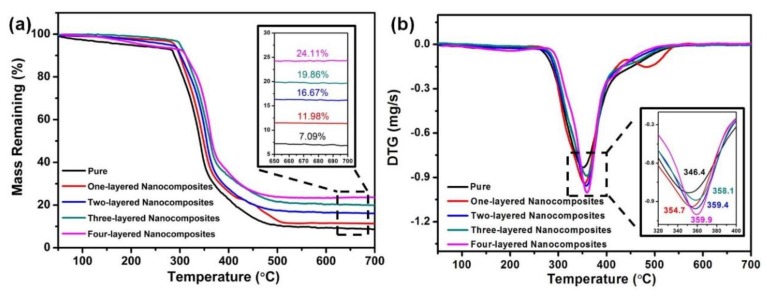
(**a**) Typical TGA curves; (**b**) DTG curves of pure epoxy polymer and CMC/buckypaper/epoxy nanocomposites.

The effects of different self-assembled CMC/buckypaper layers on the thermal stability of polymer nanocomposites to the neat epoxy resin polymer were tested through TGA experiments. Figure 8a,b shows the TGA and DTG curves of pure epoxy resin and CMC/buckypaper nanocomposites, respectively. The pure epoxy resin polymer curve in Figure 8a shows a single weight loss on the decomposition step, which was primarily attributed to the random chain scission of epoxy resin polymer chains, and this single stage progress may be caused by decarboxylation, decarbonylation, and dehydration disproportionation, cyclization, and depolymerisation. Figure 8a also indicates the pure epoxy resin polymer begins to degrade at approximately 302 °C, and the maximum rate of weight loss temperature is around 350 °C, which is depicted in Figure 8b. The CMC/buckypapers/epoxy polymer nanocomposites show the same weight loss behavior as pure polymer within the same temperature range, but the degradation temperature positions consistently increased with increasing buckypaper layers (one-layered nanocomposites: 310 °C, two-layered nanocomposites: 316.4 °C, three-layered nanocomposites: 319.1 °C), and the four-layered nanocomposite sample displayed a degradation temperature of 320.1 °C, an approximate 20 °C increase compared to the pure epoxy resin polymer sample. Moreover, the peak positions of DTG also moved towards a high temperature with the addition of CMC/buckypaper layers, as Figure 8b shows. The increase in degradation temperature and maximum rate of weight loss temperature with increasing CMC/buckypaper layers indicates an improved thermal stability of nanocomposites compared to that in pure epoxy resin polymer. At 700 °C, the residual mass of neat epoxy resin polymer was about 7.09% of the initial weight and one-layered nanocomposites showed a residual mass of 11.98%, which increased after the introduction of the buckypaper. The two-layered nanocomposite had a residual mass around 16.67%, which further increased to 19.86% for three-layered nanocomposites and 24.11% for four-layered nanocomposites. From the experimental results, it could be proven that epoxy resin nanocomposites reinforced with CMC/buckypapers have a positive influence on the thermal stability and lower degradation rate compared to pure polymer. This is a consequence of the high thermal conductivity of carbon nanotubes, which can efficiently enhance the heat transport from polymer to CNT to polymer [26]. In addition, dispersion is a key parameter deciding the thermal stability performance of polymer composites with particle fillers. Buckypaper-based composites can avoid nanotube agglomeration to achieve a better dispersion, a much higher concentration of CNTs, and high continuous thermal conductive networks for heat transport [27]. Moreover, CMC/buckypapers made with CNTs have an extremely high surface area that may hinder polymer degradation due to molecular interactions and mechanical interlocking between buckypapers and polymer chains near the nanotube molecular surface, which may noticeably decrease molecular chain mobility, hence slowing down the decomposition process [28,29].

**Figure 9:**
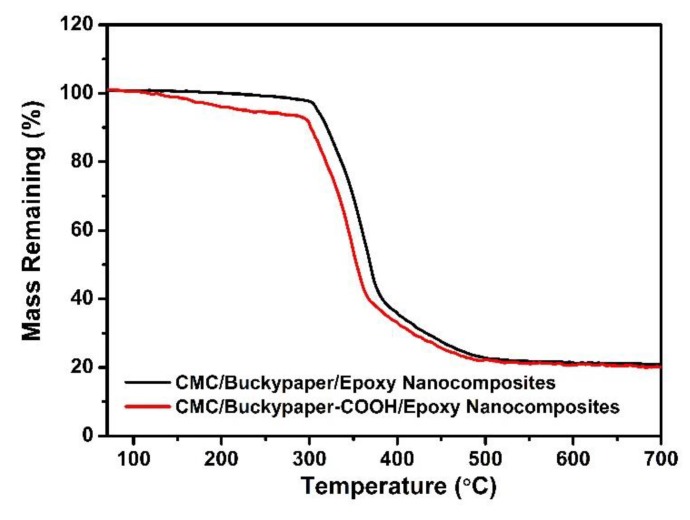
Typical TGA curves of unmodified and modified CMC/buckypaper/epoxy nanocomposites.

Further tests were carried out to characterize the effect of the carboxy-modified CNTs on the calorimetric behavior of CMC/buckypaper/epoxy nanocomposites, as shown in Figure 9. Although the residual mass of CMC/buckypaper/epoxy nanocomposites is bigger than that of neat polymer, the values are almost the same for CMC/buckypaper/epoxy nanocomposites and CMC/buckypaper–COOH/epoxy nanocomposites. It is interesting to highlight that the maximum rate of weight loss temperature of functionalized-buckypaper-reinforced polymer nanocomposites is shown to be a little lower than that of buckypaper nanocomposite. Moreover, the CMC/buckypaper/epoxy nanocomposites made with functionalized CNTs begin to degrade after approximately 100 °C. In all probability, such a significant degradation was brought forth by the degradation of the carboxyl groups. This is also owing to the fact that pristine CNTs are more thermally stable in comparison to the functionalized ones due to their defect-free, crystalline structure. According to the investigation of TGA, CMC/buckypaper/epoxy nanocomposites are very stable under 200 °C and could be used as high-temperature-resistant materials.

### 3.7. Dynamic Mechanical Performance of CMC/Buckypaper/Epoxy Nanocomposites

**Figure 10:**
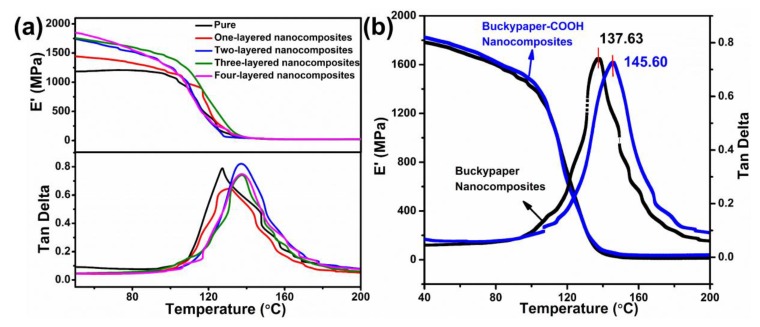
Temperature-dependent dynamic–mechanical data of the investigated samples. (**a**) Storage modulus plots and tan d plots of different CMC/buckypaper layers nanocomposites; (**b**) storage modulus plots and tan d plots of unmodified and modified three-layered CMC/buckypaper nanocomposites.

In terms of mechanical performance, CMC/buckypapers have good strength retention at high temperatures, which was further confirmed through dynamic mechanical analysis (DMA). For neat epoxy and CMC/buckypaper/epoxy polymer nanocomposites, the data plots for storage modulus and dissipation factor (tan d) versus temperature are presented in Figure 10. It was found that the storage modulus of each sample below the glass transition temperature (Tg) was about two orders of magnitude larger than that above the Tg value. For instance, the three-layered buckypaper nanocomposite exhibited a storage modulus of 1821 MPa at room temperature, while it is just 57 MPa at 140 °C. In the meantime, the storage modulus of CMC/buckypaper/epoxy polymer nanocomposites was dramatically improved with the incorporation of different CMC/buckypaper layers over most of the temperature ranges tested. The nanocomposite with four-layered CMC/buckypaper possessed the highest storage modulus of 1934 MPa, which was approximately 60% higher than that of neat polymer (1185 MPa) below the range of glass transition region, whereas there were no significant differences in the rubbery plateau region. This increased storage modulus is attributed to the ultrahigh elastic modulus of CNTs (over 1 TPa) [30,31] and the effect of the homogeneous CMC/buckypapers in an epoxy matrix coupled with significant carbon nanotube/polymer interactions, resulting in the effective knotting and tangling between CNTs and epoxy matrix chains [32]. The glass transition temperature (Tg) of neat epoxy polymer is 130.9 °C, determined from the peak position of the tan d curves. It is interesting to highlight that a further slight improvement of Tg is obtained as well after introducing more than two layers of CMC/buckypaper into epoxy polymer, demonstrating that the heat resistance ability of nanocomposites increased. The Tg of two-layered CMC/buckypaper/epoxy nanocomposites is 137.11 °C, which is 6.21 °C higher than that of pure epoxy resin, and that of nanocomposites fabricated using three and four layers CMC/buckypaper is 137.63 °C and 137.52 °C, respectively. Moreover, the height of the tan d peak for CMC/buckypaper/epoxy nanocomposites is slightly lower than that of the neat epoxy polymer, implying a low energy dissipation of CMC/buckypaper/epoxy nanocomposites. This modest enhancement could also reveal strong interactions between CNTs and polymer molecules; the presence of CNTs hinders the segmental motions of polymer chains by mechanical interlocking and helps polymers to absorb and transfer external force effectively [33]. As a result, there was an improvement in the mechanical properties and the CMC/buckypaper/epoxy nanocomposites exhibited a lower height value of tan d compared to that of epoxy resin.

Regarding the effect of the carboxy-modified CNTs on the mechanical property of CMC/buckypaper/epoxy polymer nanocomposites, the nanocomposites made with modified CMC/buckypapers were investigated; the results are shown in Figure 10b. No significant variation could be obtained in the storage modulus and the height of tan d peak; the storage modulus value of the unmodified and modified samples is 1821 and 1844 MPa, respectively. Compared to unmodified CMC/buckypaper nanocomposites, CMC/buckypaper–COOH nanocomposites showed an increase of 7.97 °C when there were three CMC/buckypaper layers. The difference in Tg may be attributed to the strong interfacial bond and good dispersibility of modified CNTs reducing the mobility of epoxy molecules and resulting in increased thermal stability [34]. The excellent thermal mechanical performance shows that CMC/buckypaper/epoxy nanocomposites are a promising material for high-temperature-resistant material applications, such as wide-temperature-range aerospace and fire-resistant and avionic power conditioning capacitors.

## 4. Conclusions

In conclusion, a high electroconductive self-assembled carbon nanotube paper has been successfully fabricated through vacuum filtration, using CMC as a binder. The self-assembled CMC/buckypapers presented a uniform self-supported network structure and excellent electrical conductivity. The lowest resistivity could reach 0.43 ± 0.03 Ω·cm, when buckypaper was prepared by the same mass of CMC and carboxy modification of carbon nanotubes. CMC helped carbon nanotubes to form a large and stable network, and these networks provided paths for the electron transfer, which led to the reduction of the intertube contact resistance. Nanocomposite materials were manufactured by self-assembled CMC/buckypapers as the reinforcement and epoxy resin as the matrix through the resin impregnation molding technique. CMC/buckypaper/epoxy nanocomposites made with carboxylated carbon nanotubes had the lowest resistivity (4.75 ± 0.15 Ω·cm) and highest bending strength (125.04 ± 5.62 MPa) and bending modulus (5.83 ± 0.68 GPa) when four layers of CMC/buckypaper were put into epoxy resin polymer. The corresponding experiments (DMA and TGA) were carried out to evaluate the effect of self-assembled CMC/buckypapers on the heat and thermomechanical properties of epoxy nanocomposite materials. CMC/buckypapers have a positive influence on the thermal stability and lower degradation rate compared to pure polymer; the storage modulus and glass-transition temperature were also improved by increasing layers of CMC/buckypapers. Overall, the self-assembled CMC/buckypapers which exhibit a stabilized electrical conductivity could be used as an antistatic packaging material, transducer, electrode, and capacitor. Moreover, the epoxy nanocomposites made with self-assembled CMC/buckypapers that showed good mechanical properties and an excellent thermal and thermomachanial performance are a promising material for high-temperature-resistant material applications.

## Figures and Tables

**Table 1:** Main parameters of the characteristic Raman spectras.

Samples	The Centre of D Band/cm^−1^	FWHM/cm^−1^	I_D_/Count	The Centre of G Band/cm^−1^	FWHM/cm^−1^	I_G_/Count	I_D_/I_G_
Buckypaper	1336	34.97	21572.79	1585	61.93	42830.94	0.50
CMC/Buckypaper	1343	37.03	43067.93	1585	66.55	73135.80	0.59
Buckypaper-COOH	1351	56.36	59230.92	1587	60.71	137469.99	0.43
CMC/Buckypaper-COOH	1344	64.01	261193.85	1582	69.84	217418.57	1.20
